# Electroporation by nucleofector is the best nonviral transfection technique in human endothelial and smooth muscle cells

**DOI:** 10.1186/1479-0556-3-2

**Published:** 2005-04-18

**Authors:** Nina Iversen, Baard Birkenes, Kari Torsdalen, Srdjan Djurovic

**Affiliations:** 1 Department of Medical Genetics, Ullevål University Hospital, Oslo, Norway

**Keywords:** Electroporation, Gene Therapy, Liposomes, Lipofection, Photochemical Internalization, Nucleofection, Transfection

## Abstract

**Background:**

The aim of this study was to determine the optimal non-viral transfection method for use in human smooth muscle cells (SMC) and endothelial cells (EC).

**Methods:**

Coronary Artery (CoA) and Aortic (Ao) SMC and EC were transfected with a reporter plasmid, encoding chloramphenicol acetyltransferase type 1 (CAT), with seven different transfection reagents, two electroporation methods and a photochemical internalization (PCI) method. CAT determination provided information regarding transfection efficiency and total protein measurement was used to reflect the toxicity of each method.

**Results:**

Electroporation via the nucleofector machine was the most effective method tested. It exhibited a 10 to 20 fold (for SMC and EC, respectively) increase in transfection efficiency in comparison to the lipofection method combined with acceptable toxicity. FuGene 6 and Lipofectamine PLUS were the preferred transfection reagents tested and resulted in 2 to 60 fold higher transfection efficiency in comparison to the PCI which was the least effective method.

**Conclusion:**

This study indicates that electroporation via the nucleofector machine is the preferred non-viral method for *in vitro *transfection of both human aortic and coronary artery SMC and EC. It may be very useful in gene expression studies in the field of vascular biology. Through improved gene transfer, non-viral transfer techniques may also play an increasingly important role in delivering genes to SMC and EC in relevant disease states.

## Background

Several methods have been described to introduce DNA expression vectors into mammalian cells *in vitro *and *in vivo*: calcium phosphate precipitation, microinjection, electroporation, receptor-mediated gene transfer, particle guns, viral vectors, and lipofection [1-3]. Each system has benefits and limitations, and to date there is no ideal method for gene transfer.

Viral vector systems, derived from modified animal or human viruses, resulting in replication-deficient vectors [4], represent a powerful transfection tool. Nevertheless, their immunogenicity, oncogenic properties, inactivation of vector, development of replication-competent virions and need for a relatively large-scale infrastructure for their production are serious disadvantages [5].

The use of cationic liposome/DNA complexes (lipofection) for gene transfer into somatic cells has become a popular method of delivering genes. Interaction between cationic lipids and DNA through ionic interaction leads to forming cationic lipoplexes [1,4]. The resulting complexes fuse with the anionic surfaces of cells, delivering DNA into the cells via endocytosis. However, the final transport of DNA into the nucleus is still not fully understood. Although inferior, transfection using lipofection offers some advantages over viral vectors, such as simplicity of production, low toxicity and low immunogenicity.

Another transfection method, electroporation [6], also termed electrotransfer [7] or electropermeabilization [8], is an experimental technique involving the application of brief electric pulses to cells or tissues in order to increase cellular permeability to macromolecules. This method has been reported to increase naked DNA expression by 100-fold or more [6-8]. Finding the balance between the best possible transfection efficiency and survival rate is very important, therefore we investigated the optimization of this technique using two different electroporation instruments.

Photochemical internalization (PCI) was reported as a procedure for site-specific delivery of several types of membrane impermeable macromolecules from endocytotic vesicles to the cytosol [9]. This technology is based on the cytosolic release of endocytosed macromolecules from endosomes and lysosomes which become localized to these vesicles upon exposure of cells to photosensitizing compounds and light. PCI has several advantages over other conventional applications for the cytosol delivery of membrane impermeable molecules [10]. One advantage is that there are no restrictions on the type and size of the molecule to be internalized, as long as the molecule of interest can be endocytosed. We examined the applicability of PCI technology to our cells of interest.

In this study we present extensive investigations performed with transfection reagent mediated transfections, electroporation and PCI. The aims of the study were to evaluate the efficiency and safety of optimized novel non-viral transfection techniques for our four cell types of interest: coronary artery (CoA) SMC, aortic (Ao) SMC, CoAEC and AoEC. Our results showed that electroporation via the nucleofector machine turned out to be the most effective non-viral method for *in vitro *transfection of both human SMC and EC, while FuGene6 and Lipofectamine PLUS appeared as best performing lipofection reagents. These results also provided useful informations regarding optimization and selection of transfection conditions for the cell types tested.

## Methods

### Cell cultures

Human Coronary Artery (#CC-2583) and Aortic (#CC-2571) SMC were obtained from Clonetics Corporation (Walkersville, MD) together with human Coronary Artery (#CC-2585) and Aortic [#CC-2535] EC. The cells had been isolated from normal human tissue and cryopreserved in smooth muscle cell media, SmGM-2 (#CC-3182) and endothelial cell media, EGM-2-MV (#CC-3202) respectively, supplemented with 10% FCS (Gibco BRL, Gathersburg, MD) and 10% dimethyl sulfoxide in order to improve cell viability and seeding efficiency upon thawing. Cells were cultivated in modified Sm basal medium (SmBM; #CC-3181) supplemented with SmGM-2 Single Quots and growth factors (#CC-4149) or, for EC in EBM-2 basal medium (#CC-3156) supplemented with EGM-2-MV Single Quots and growth factors (#CC-4147) (Clonetics Corporation, Walkersville, MD) and 5% FCS. Cells were incubated at 37°C in a humidified atmosphere with 95% air and 5% CO_2_. Medium was changed every second day and the protocols from producer were strictly followed. For the transfection experiments, low-passage cells (passages 4 to 8) at 80% confluency were used.

### Plasmid vectors

The bacterial enzyme, CAT, encoded by Tn9, has no eukaryotic equivalent and has become one of the standard markers used in transfection experiments.

The pRc/CMV2/CAT plasmid supplied by Invitrogen (Carlsbad, CA, USA) was used in this study. We amplified the plasmid using competent E. coli cells from One Shot chemical transformation kits supplied by Invitrogen (Carlsbad, CA, USA). Bacteria were grown and the plasmid was isolated using GigaPrep kit, QIAGEN (Valencia, CA, USA).

### Transfection reagents

Seven commercially available transfection reagents were used:

• FuGENE 6 (Roche, Mannheim, Germany), a non-liposomal transfection reagent, proprietary blend of lipids and other compounds,

• Lipofectamine PLUS (Invitrogen, Carlsbad, CA, USA), a 3:1 liposome formulation of the polycationic lipid 2,3-dioleyloxy-N(2(sperminecarboxamido)ethyl)-N,N-dimethyl-1-propanaminium trifluoroacetate (DOSPA) and the neutral lipid dioleoyl phosphatidylethanolamine (DOPE) in membrane-filtered water. PLUS reagent is used to pre-complex DNA prior to the preparation of the transfection complexes,

• Metafectene (Biontex, Munich, Germany), a polycationic transfection reagent that encompasses "repulsive membrane acidolysis" which ensures destabilization of the DNA-coating lipid membrane by repulsive electrostatic forces in the weakly endosomal acidic environment and release of the DNA into the cell protoplasm,

• Lipofectamine 2000 (Invitrogen, Carlsbad, CA, USA), a cationic lipid that allows high transfection efficiencies and protein expression levels,

• GenePORTER (Gene Therapy Systems Inc., San Diego, CA, USA), a formulation of the neutral lipid DOPE and a proprietary cationic lipid derived from hydrophilic conjugation technology,

• LipoGen (InvivoGen, San Diego, CA, USA), a formulation of a unique lipid that combines in its structure the characteristics of both a cationic lipid and a fusogenic lipid, such as DOPE, which works via the unsaturated hydrocarbon chains of DOPE which destabilize membrane bilayers, thereby facilitating delivery of lipid/DNA complexes into the cells, and

• Lipofectin (Invitrogen, Carlsbad, CA, USA) a 1:1 liposome1 liposome formulation of cationic lipid N-(1-(2,3-dioleyloxy)propyl) -n,n,n-trimethylammonium chloride (DOTMA) and DOPE in membrane filtered water.

### Transfection by reagents

Low-passage cells were cultivated and used in 6-well plates 18 h before transfection. Approximately 3 × 10^5 ^cells per well (80% confluence) were used in transfections.

The transfections, using reporter vector complexed with each of the tested reagents, were performed according to the manufacturer's protocols.

Plasmid DNA (0.8–6 μg CAT) at different DNA:liposome ratios (1:3 – 1:5) was diluted in separate tubes containing 100 μl – 1000 μl of serum-free media, mixed and incubated 15–45 min at room temperature. Media was removed and transfection solutions were added to each well (100 μl – 1000 μl). After 3 – 6 hrs incubation at 37°C and 5% CO_2, _1 ml fresh media (with FCS and supplements) was added to each well and transfection continued for 24 hours.

### Transfection by electroporation

Two different methods of electroporation were tested, each using a different instrument. Firstly, electroporation was conducted with ECM 630 electroporator (BTX, San Diego, CA, USA) and secondly, the nucleofector instrument, (Amaxa Biosystems, Cologne, Germany) was tested.

Cells were grown in T175 bottles, trypsinized, collected by centrifugation (200 × g, 10 min) and resuspended in medium containing 10% FCS for EC and Hanks solution for SMC. 0.4 ml containing approximately 2 × 10^6 ^cells and 20 μg CAT plasmid (1 μg/μl) was placed in a sterile electroporation cuvette (BTX 0,2 cm gap). Cells were subjected to high-voltage at a setting that had been optimized for each cell type. After electroporation, the cells were immediately plated out using pre-warmed growth media supplemented with 10% FCS in 6 well plates.

For transfection with the Nuclefector instrument, a specific optimized electroporation method and a specific nucleofector solution were used for each cell type. For SMC the human AoSMC Nucleofector™ kit was used (VPC-1001). Cells were grown in T175 bottles, trypsinized, collected by centrifugation (200 × g, 10 minutes) and resuspended in the HCAEC nucleofector solution at two cell suspensions of 5 × 10^5 ^and 1 × 10^6 ^cells per 100 μl and 1–10 μg DNA (1 μg/μl CAT). Program U-25 was applied. For CoAEC the human HCAEC Nucleofector™ kit (VPB-1001) was used. CoAEC were treated as SMC, except that they were tested at a single concentration of 5 × 10^5 ^cells per 100 μl. 100 μl of cell suspension and 1–10 μg DNA (1 μg/μl CAT) were mixed and transferred to a cuvette. Program S-05 was used. After treatment, the cells were immediately plated out in pre-warmed medium, supplemented with 10% FCS, into 6 well plates.

### Transfection by photochemical internalization (PCI)

Photochemical internalization was conducted with a LumiSource™ (PCI Biotech AS, Oslo, Norway). Reagents (LumiTrans and p(Lys)) were also provided from PCI Biotech.

For this method 7 × 10^4 ^were cells plated into 12-well culture plates. The next day media was removed and the cells were treated with 0.4 ml of the photosensitizer LumiTrans in medium containing 10% FCS (2 μg/ml) for 16–18 hours at 37°C. The cells were washed three times with medium. For Optimization of light dose, 0.8 ml fresh medium was added to cells before exposure to the LumiSource for 20 to 200 sec. Cell lysates were harvested after 24 hours and total protein measurement was carried out. The light dose that gave 50% survival was set as the highest dose and a range of lower light doses was used for optimization of the PCI method.

#### Photochemical transfection

Plasmid-p(Lys) complexes were formed by gentle mixing of 75 μl cell suspension with 2–20 μg CAT plasmid (1 μg/μl), water with 5.35 μl of p(Lys) (1 μg/μl) and 69.65 μl of water. The resulting solution was incubated for 30 minutes at room temperature before being diluted to 1 ml with medium. Cells were incubated with 0.4 ml of the plasmid mixture for 4 hours at 37°C. When the cells were washed once with medium, fresh medium (0.8 ml) was added and the cells were exposed to LumiSource light doses. The cells were exposed to increasing light doses before the transfection.

### Post-transfection cell treatment

24 hrs after transfection, media was removed and cells were washed 3 times with 1 × PBS and lysed in 1 or 2 ml CAT lysis buffer (supplied in CAT ELISA kit, Roche, Mannheim). Cell lysates were used for CAT determination and total protein measurement assay.

### CAT ELISA Measurements

Concentrations of CAT in cell lysate were measured by CAT-ELISA (Roche, Mannheim, Germany) as recommended by the producer. All measurements were done in duplicate and concentrations of unknowns were determined from standards run with each plate.

### Cell Survival calculations

Cellular total protein was measured by an improved Lowry assay (Bio-Rad *D*_*C *_Protein Assay, Bio-Rad Laboratories, Hercules, CA, USA). When comparing the results from test and control wells, it was assumed that cells in the control well were unaffected by the experiment. Test results were then compared to the control results and a percentage survival was calculated.

These measurements were confirmed using a Cytotoxicity Detection Kit (Roche, Mannheim), which measures lactate dehydrogenase (LDH) activity released from damaged cells (results not shown).

### Reporting of results

In order to effectively compare the results from each of the three methods, we standardized the results according to the number of cells used : transfection reagents requiring only 3 × 10^5 ^cells while electroporation and PCI use 1–2 × 10^6 ^cells per well. To standardize, we used a ratio of CAT produced (ng) divided by total protein of surviving cells (ng), thereafter called transfection efficiency (the amount of CAT produced per living cell). This value was then multiplied by 1 × 10^6 ^to make the numbers more manageable. This calculation does not take into account the differences in cell survival, and that is why this should be considered as well. for the comparisons of transfection efficiency

## Results

### Transfection by reagents

**Figure 1:**
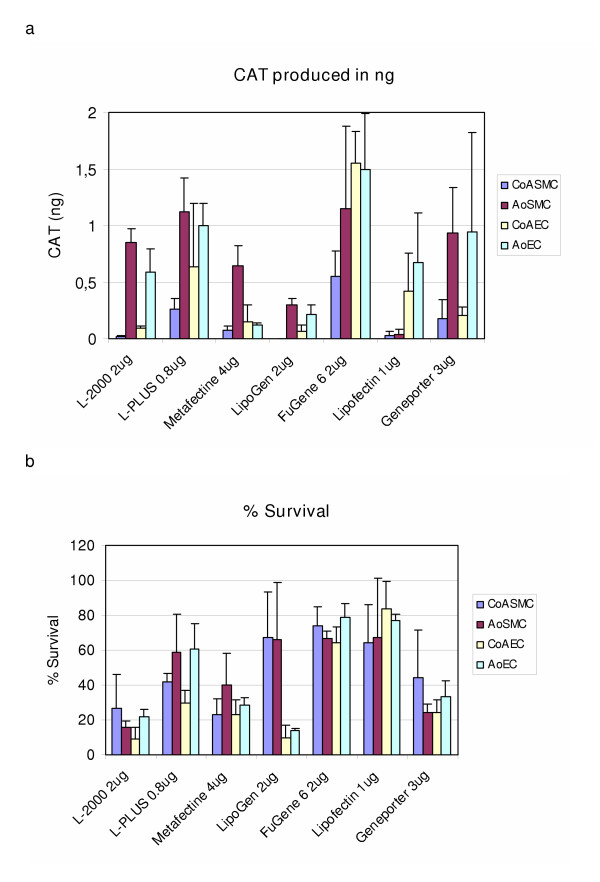
Figure (a) shows the amount of chloramphenicol acetyltransferase (CAT) produced in each of the cell lines, when the different transfection reagents were used, at optimal plasmid amount. Figure (b) shows the corresponding % survival when each of these reagents and plasmid amounts were used. Note: Results are shown as a mean +/- SD of two individual experiments (performed in duplicate).

In order to determine the preferred transfection reagent for each cell type, we comparatively considered the following: the amount of CAT produced, the ratio between CAT/total protein and the cell survival. When considering the results obtained in the four cell types used, the results show that the three best performing reagents were FuGENE 6, Lipofectamine PLUS and GenePORTER (Table 1 and Figure 1). As presented in Table 1, the values display a range across the four cell types used. Individual results are reported in the text below and in Figure 1, where the results found using the optimal concentration of plasmid for each reagent are displayed.

**Table 1:** Summary of the results obtained from cells transfected with chloramphenicol acetyl transferase using the seven different transfection reagents tested. Results are given as a range across all cell types.

**Liposome**	**Manufacturer**	**DNA amount**	**Liposome: DNA ratio**	**Transfection Efficiency**	**% Cell Survival**
FuGENE 6	Roche	1 and 2 μg	3:1	3.0 – 16.4	65 – 80
Lipofectamine 2000	Invitrogen	2 and 4 μg	3:1	0.4 – 34.0	9 – 27
Lipofectamine PLUS	Invitrogen	0.8 and 1.6 μg	3:1	3.4 – 18.3	18 – 61
Metafectene	Biontex	2 and 4 μg	3:1	0.6 – 8.6	25 – 50
Lipofectin	Invitrogen	1 and 2 μg	4:1	0.0 – 7.1	65 – 100
GenePORTER	Gene Therapy Solutions	3 and 6 μg	5:1	1.6 – 21.9	24 – 55
LipoGen	Invivo	1 and 2 μg	3:1	0.0 – 13.9	10 – 90

In CoASMC, use of FuGENE 6 achieved the best results. It produced almost twice as much CAT per ml media than cells transfected using the second best performing reagent, Lipofectamine PLUS (Figure 1). When 1 μg and 2 μg plasmid were used, ratios of 3–5 were obtained and the cell survival rate was between 69 and 74%, respectively (Figure 1). In AoSMC, Lipofectamine PLUS gave the best results. It produced more CAT per ml media than cells transfected with the next best performing reagent, FuGENE 6 (Figure 1). When 0.8 μg and 1.6 μg plasmid was used, ratios of 10–18 were obtained and the cell survival rate was between 53 and 58%, respectively (Figure 1).

In CoAEC, best transfection efficiency was achieved by FuGENE 6 : it produced more than double the amount of CAT per ml than cells transfected using the other reagents (Figure 1). When 1 μg and 2 μg of plasmid were used, ratios of 8–11 were obtained and the cell survival rate was between 64 and 73%, respectively (Figure 1).

FuGENE 6 gave the best results in AoEC, as well : it produced more CAT per ml media than cells transfected with the second best liposome, Lipofectamine PLUS (Figure 1). When 1 μg and 2 μg of plasmid was used, ratios of 7–16 were obtained and the cell survival rate was between 79 and 88%, respectively (Figure 1).

### Transfection by electroporation

#### Electroporator

**Figure 2:**
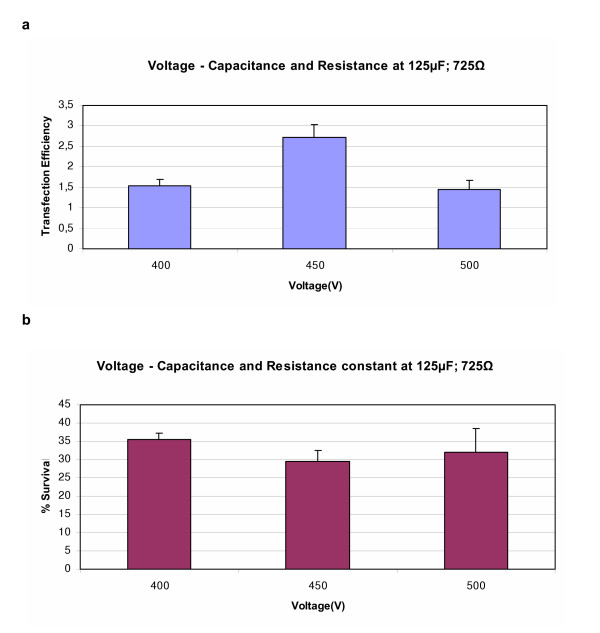
Figure (a) shows the transfection efficiency obtained in AoSMC when voltage settings were varied using the ECM 630 electroporator. Capacitance and Resistance were held constant at 125 μF and 725Ω respectively. Figure (b) shows the % survival obtained at the corresponding settings. Results represent mean of triplicates +/- SD of a typical experiment.

To optimize the electroporation procedure, a range of voltage, capacitance and resistance settings were used. For SMC the initial resistance and capacitance settings were 725Ω and 125 μF and for EC they were 950Ω and 25 μF. The voltage settings tested varied from 400 – 500 V. The optimal voltage in all four cell types was 450 V, illustrated by AoSMC (Figure 2).

**Figure 3:**
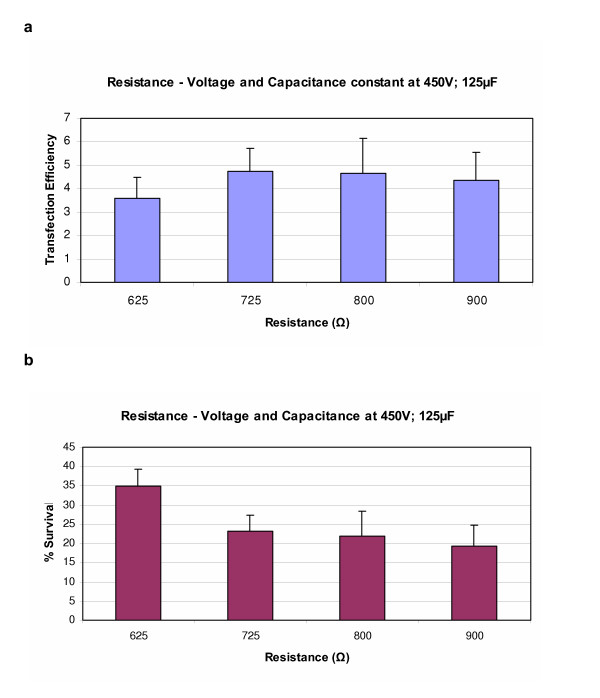
Figure (a) shows the transfection efficiency obtained in AoSMC when resistance settings were varied using the ECM 630 electroporator. Voltage and capacitance were held constant at 450 V and 125 μF respectively. Figure (b) shows the % survival obtained at the corresponding settings. Results represent mean of triplicates +/- SD of a typical experiment.

**Figure 4:**
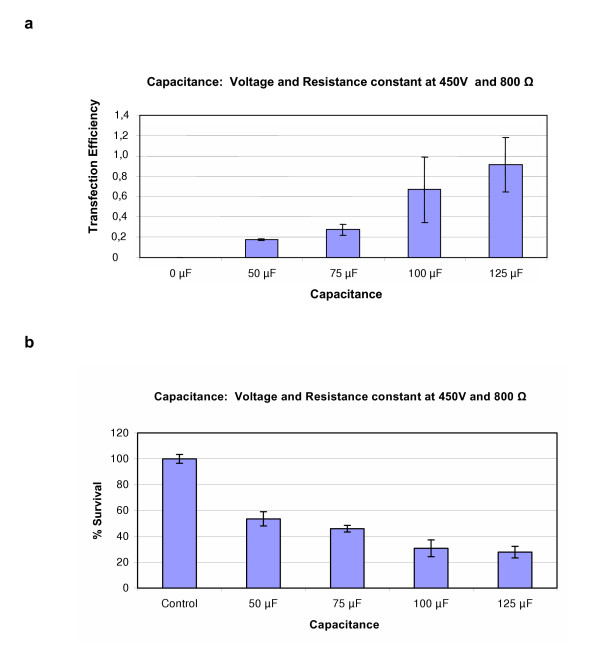
Figure (a) shows the transfection efficiency obtained in AoSMC when different capacitance settings were used on the ECM 630 electroporator (BTX, San Diego, USA). Voltage and resistance were held constant at 450 V and 800Ω, respectively. Figure (b) shows the % survival obtained at the corresponding settings. Results represent mean of triplicates +/- SD of a typical experiment.

After the voltage settings had been established the optimal resistance and capacitance were found. For CoA and Ao SMC the best resistance setting was found to be in the area 725–900Ω (Figure 3), but best capacitance varied between the two cell types. In CoASMC, the best capacitance setting was 75 μF (Ratio 2.5 and 70% survival). In some experiments, we achieved a ratio of up to 6 when 125 μF was used, but survival dropped to around 30%. Nevertheless, we choose 75 μF as the best setting because it resulted in higher cell survival. In AoSMC the best results were obtained when 125 μF were used (Ratio of 0.92 and a survival of 30%) (Figure 4). The higher the capacitance settings was, the lower become the cell survival (Figure 4b).

Both CoA and Ao EC reacted similarly to the different settings. Resistance was tested between 850–1050Ω and at 900Ω a ratio of 25 was obtained (55% survival). We tested capacitance varying from 25 – 75 μF. When 50 μF was used, we obtained a ratio of 40 and a survival of 38%. However, 25 μF was the best setting since it resulted in better cell survival (61%) (results not shown).

#### Nucleofector

**Figure 5:**
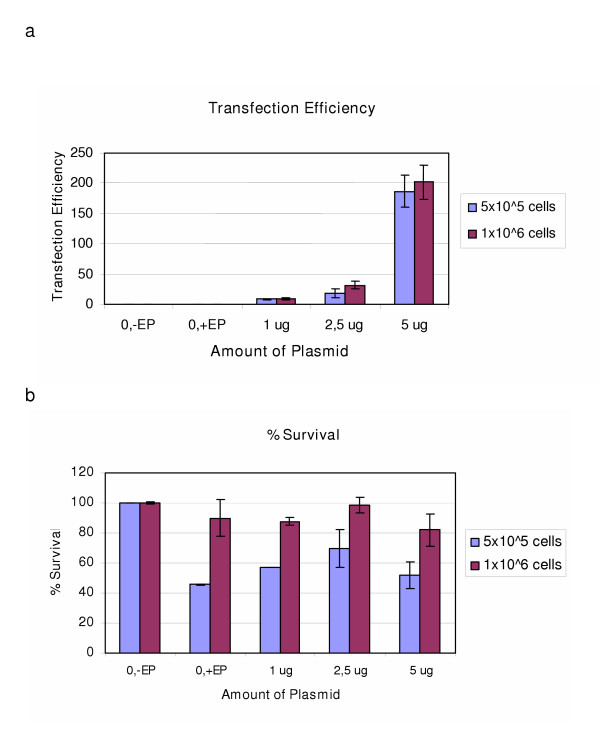
Figure (a) shows the transfection efficiency obtained in AoSMC when different amounts of CAT plasmid were transfected into different cell numbers using the Nucleofector instrument, program U-25. Figure (b) shows the % survival obtained at the corresponding plasmid amounts. Results represent mean of duplicates +/- SD.

Optimized nucleofector protocols were available for AoSMC and CoAEC. These methods were tested and the results were compared with the electroporation results. For AoSMC we tested two cell suspensions, 5 × 10^5 ^and 1 × 10^6^cells per reaction. Both the ratio and the survival increased by increasing the number of cells used (Figure 5a). At the highest plasmid dose, the cell survival was 80% (Figure 5b).

**Figure 6:**
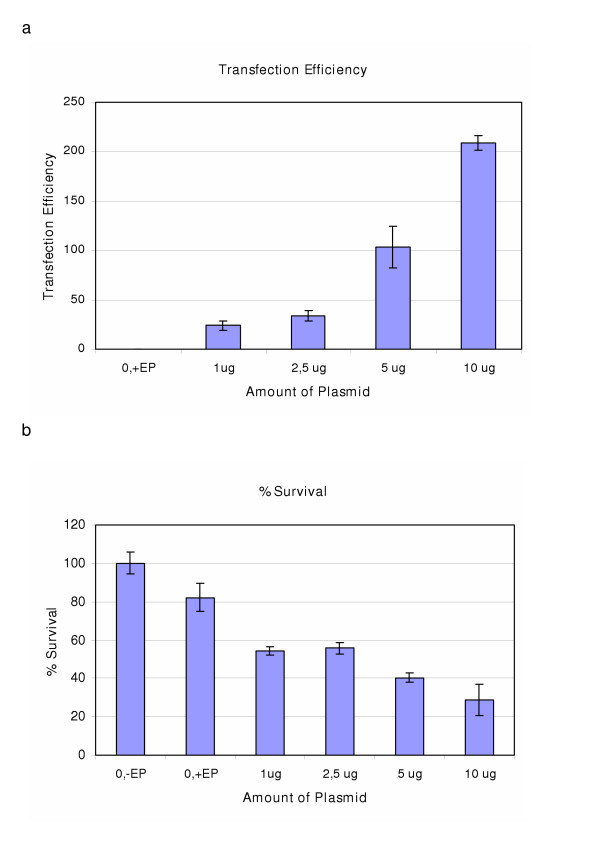
(a) and (b): Figure (a) shows the transfection efficiency obtained in CoAEC when different amounts of CAT plasmid were transfected, using the Nucleofector instrument (Amaxa Biosystems, Cologne, Germany), program S-05. Figure (b) shows the % survival at the corresponding plasmid amounts. Results represent mean of triplicates +/- SD of a typical experiment.

In CoAEC, we observed a dose-response for the CAT/protein ratio when 1–10 μg plasmid was used (Figure 6a), and at the highest plasmid dose of 10 μg, 30–46 % cell survival was achieved (Figure 6b).

### Transfection by PCI

The initial experiments with PCI were aimed to find the light dose at which we obtained at least 50 % survival. For AoSMC this was observed to be 100 sec. In further experiments light doses varying from 25 to 100 seconds were used. A low transfection effect, ratio of 0.3, was achieved when the cells were exposed to light before the transfection of 5 μg plasmid (Table 2).

**Table 2:** The best results obtained in AoSMC and CoAEC when different transfection methods were used.

**Methods**	**Best Transfection Efficiency**		**Corresponding % Cell Survival**	
	**SMC**	**EC**	**SMC**	**EC**
Transfection Reagent	18	11	53	74
ECM 630	5.5	25	38	55
Nucleofector	200	209	80	30
PCI	0.3	4.7	84	55

The light dose that gave 50% survival in CoAEC was between 40 and 50 seconds, and for AoEC it was 32 seconds. The best transfection effect obtained had a ratio of 4.7 and 55% survival, when the cells were given 5 μg plasmid before exposure to light for 25 seconds (Table 2). None effect was seen when the cells were exposed to light after addition of plasmid.

## Discussion

Improvement of the delivery efficiency of genes into SMC and EC and the development and optimization of transfection methods has increasingly become an important research objective. In this study we found that transfection by electroporation, using the nucleofector instrument, was comparatively the most effective transfection method combining both high efficiency and acceptable survival rate for both smooth muscles cells and endothelial cells (Table 2). Enhancement of transfection efficiency by transfection reagents and the ECM 630 instrument also worked well, but not to the same extent as nucleofection (Table 2).

Transfection using the nucleofector is a patented commercial technique requiring special buffers and programs, the constituents of which are a secret. Nevertheless, we developed "in house" methods for ECM 630 electroporator machine. Optimizing these methods is possible, but many variables have to be taken into account. In this study we used constant buffer, cell numbers and plasmid amounts in order to test and optimize the variables available on the instrument (voltage, capacitance and resistance). From our findings we can conclude that transfer efficiencies could be greatly improved. We believe that electroporation by nucleofection is an easy and effective method for transfecting human EC and SMC, although the high number of cells and high plasmid amounts required could be considered a weakness.

On the other hand, the use of transfection reagents in *in vitro *cell transfection is easy, affordable and requires low cell numbers. Enhancement of transfer efficiency was achieved by reagents in all the cell types tested. However, the best performing transfection reagents found in certain cell types are not necessarily the optimal reagents for other cell types. Nevertheless, FuGENE 6 reagent has shown similar transfection efficiency across all four cell types. Of further importance is to notice that results in this study reflect those setups recommended by each reagent manufacturer. As expected, optimization of transfection condition by these reagents may also lead to an improvement in transfection efficiencies. For optimal transfer efficiencies, higher DNA concentrations require higher amounts of liposomes, which will inevitably increase cell death. FuGENE 6 achieved relatively good transfer efficiencies combined with low toxicities.

Another transfection reagent, GenePORTER, demonstrated high transfer efficiencies, although accompanied with relatively high toxicity. This may be attributed to GenePORTER requirements for high amounts of DNA and liposome to be successful.

Generally, the observed differences in the transfer efficiency and the optimal DNA/liposome ratios may depend on the readiness of cells to take up DNA/liposome complexes [11-14]. Our findings suggest that the ratios could not be generalized, and they have to be specified for each cell type and liposome used. The negative polarity of the cell surface appears to play a key role in the process. Therefore, transfection using reagents/liposomes needs to be optimized for each targeted cell type and reagent used [15].

PCI was yet another method we tested, but considering comparatively lower transfer efficiency, the time-consuming and demanding procedure, this method could not be evaluated as the most suitable one. It should be noted though, that PCI has shown promising applications in cancer therapy [9].

Several studies have reported optimization of non-viral gene transfer techniques to individual cells [16-18]. Up to our knowledge, this is the first report that compared lipofection, electroporation and PCI at the same experimental settings on human Ao and CoA SMC and EC.

Electroporation and PCI may prove difficult to use in a clinical setting. Regarding electroporation, tissues would have to be electroporated by using methods that are yet not well established. It has been reported [19,20] that *in vivo *electroporation has been implicated as the major cause of muscle damage in studies with electrical trauma. Moreover, clinical use of this method resulted in transient to permanent alterations in membrane permeability [19,20]. Furthermore, the extent of muscle damage may depend on pulsing parameters and electrode design [8,21-23]. Hartikka *et al*. [24] reported extensive lesions containing necrotic myofibers and heavily populated with infiltrating inflammatory cells.

Through improved gene transfer, non-viral therapeutic techniques may play an increasingly important role in delivering genes to cells in relevant disease states. One example is the cardiovascular field [25-27], and this is the reason why we tested and optimized these techniques using aortic and coronary artery SMC and EC. A strong theoretical advantage of cardiovascular gene therapy is the ease of access and, in some conditions only a temporary expression of the transfected gene is needed to achieve a beneficial biological effect [28]. Therefore, non-viral transfection techniques might offer a therapeutic option and prove suitable for the treatment of cardiovascular disease, and informations regarding optimization of transfection conditions in order to improve transfection efficiency and reduce cytotoxicity, such from our study, may be valuable for use in gene therapy studies.

## Conclusion

From the results achieved in this study it is evident that electroporation by the nucleofector instrument is the preferred transfection method for all four cardiovascular cell types. Nucleofection technique exhibited a high transfection efficiency and acceptable cell survival rate and should be very useful in gene expression studies in cardiovascular biology. As a step toward further development of gene therapy strategy, extensive *in vitro *studies with novel techniques presented within this work are essential in definition of the most suitable transfer methods.

## Competing interests

The author(s) declare that they have no competing interests.

## Authors' contributions

**N.I. **participated in the design and coordination of the study, established the transfection methods electroporation, nucleofection and PCI, and supervised the following optimalization of these methods. She was responsible for writing the manuscript. **B.B. and K.T. **were responsible for performing experiments independently, and interpretation of the results, while **SD **concieved the study, and participated in the design and coordination of it, and supervised liposomal transfection experiments.

All authors critically read and approved the final version of the manuscript.
